# LIVE: a manually curated encyclopedia of experimentally validated interactions of lncRNAs

**DOI:** 10.1093/database/baz011

**Published:** 2019-02-13

**Authors:** Gaole An, Jiaqi Sun, Chao Ren, Zhangyi Ouyang, Lingyun Zhu, Xiaochen Bo, Shaoliang Peng, Wenjie Shu

**Affiliations:** 1 Department of Biotechnology, Beijing Institute of Radiation Medicine, Beijing, China; 2 Department of Biology and Chemistry, College of Liberal Arts and Sciences, National University of Defense Technology, Changsha, Hunan, China; 3 College of Computer Science and Electronic Engineering and National Supercomputing Centre in Changsha, Hunan University, Changsha, China

## Abstract

Advances in studies of long noncoding RNAs (lncRNAs) have provided data regarding the regulatory roles of lncRNAs, which perform functional roles through interactions with other functional elements. To track the underlying relationships among lncRNAs, various databases have been developed as repositories for lncRNA data. However, the ability to comprehensively explore the diverse interactions between lncRNAs and other functional elements is limited. To this end, we developed LIVE (LncRNA Interaction Validated Encyclopaedia), an interactive resource to integrate the diverse interactions of functional elements with lncRNAs. LIVE is a manually curated database of experimentally validated interactions of lncRNAs with genes, proteins and other various functional elements. By mining publications, we constructed LIVE with the following three interaction networks: a binding interaction network, a regulation network and a disease network; then, we combined them to form a comprehensive lncRNA interaction network. The current release of LIVE contains the validated interactions of 572 lncRNAs in humans and mice with 103 proteins, 209 genes, 56 transcription factors and 194 diseases. LIVE provides an interactive interface with charts and figures to aid users in searching and browsing interactions with lncRNAs. LIVE will greatly facilitate further investigation into the regulatory roles of lncRNAs and is freely available.

## Introduction

LncRNAs play important functional roles in regulating biological and cellular processes, including proliferation, differentiation and development, through interacting with various genes, proteins and functional elements (1, 2). With the rapid expansion of research on lncRNAs, a number of databases have been developed to satisfy the demand for exploration of the regulatory roles of lncRNAs.

Unlike the early databases developed to deposit candidate lncRNAs from both experimental validation and computational prediction studies, recent databases have focused on the specific functional roles of lncRNAs. LincSNP contains single nucleotide polymorphisms (SNPs) identified in human lncRNAs and transcription factor binding sites (3). LncReg collects the validated regulatory relationships of lncRNAs (4). Lnc2Meth was developed specifically to store relationships between human lncRNAs and DNA methylation (5). LncChrom includes validated lncRNA-chromatin interactions (6). However, these databases face accuracy problems due to the high false positive rate of data from computational predictions and high-throughput experiments. Thus, there is still an urgent demand for an experimentally validated lncRNA database that has been manually curated. EVlncRNAs manually curated publications to include experimentally validated lncRNA interactions (7), but the potential interaction networks beneath publications are not fully revealed.

**Figure. 1:**
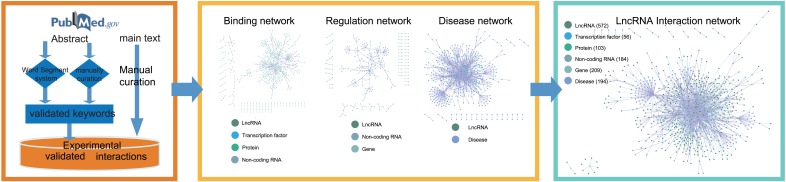
Data flow of LIVE. LIVE curated publications involved with lncRNA interactions from human and mouse to construct database.

Here we present LIVE (LncRNA Interaction Validated Encyclopaedia), a manually curated database of experimentally validated interactions between functional elements and lncRNAs from published literature available on PubMed prior to July 1, 2018. LIVE contains the validated interactions of 572 lncRNAs with 103 proteins, 209 genes, 56 transcription factors and 194 diseases in humans and mice. These validated interactions are classified into three networks, namely, a binding interaction network, a regulation network and a disease association network. By assembling these three networks, we generated a complete picture of the lncRNA interaction network with diverse types of functional regulatory elements and interactions. Moreover, an interactive interface and analysis kits are provided to help users mine the potential regulatory roles of lncRNAs in interaction networks.

## Materials and methods

To provide a comprehensive collection of experimentally validated interactions with lncRNAs, we manually curated the available publications to construct LIVE (Figure.1). First, we used `LncRNA’, `Long noncoding RNA’, and `Long ncRNA’ along with their plural forms as keywords to search the PubMed database up to July 1, 2018. The species mentioned in publications were limited to human and mouse. Second, we developed a word segment system to pre-process the abstracts and extract the keywords, including species, experiment type, disease and lncRNA. The word segment system is derived from Python module `jieba.analyse.extract_tags’ (https://pypi.org/project/jieba/), which is based on TF-IDF (term frequency—inverse document frequency) algorithm (8). TF-IDF algorithm is a statistical method used to evaluate the importance of a word to one of the documents in a document set. The importance of a word increases proportionally with the number of times it appears in a document, but decreases inversely with its frequency in the corpus. This word segment system helps extracting keywords such as disease, tissues and molecules from abstracts. Moreover, we standardized the keyword lists by comparing them with the existing databases (MeSH, HGNC, MGI and MalaCards) (9, 10). The keyword list was used to assist in describing the features of each publication by building a keyword index, which improved the efficiency and accuracy of the following manual curation. Third, we curated the abstracts to determine whether the described lncRNAs were associated with the keywords describing regulation or interaction. Fourth, we carefully curated the main text to extract the interactions and ensured that these interactions were experimentally validated. Finally, we labeled these interactions with the corresponding abstract keywords to aid the following categorization of interactions. In total, in the construction of the LIVE database, over 10 000 publications were cross-curated, and each publication was curated by at least two specialists.

**Figure. 2:**
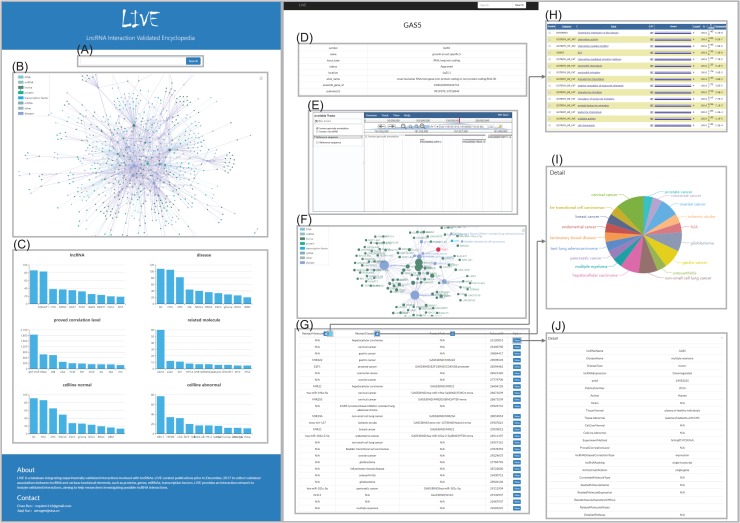
Interface of LIVE. (A–C) Main page. Search box (A), integrated network of all interactions (B) and metadata of database (C). (D–J) Detail page. Basic information of lncRNA (D), genomic browser (E), interaction network (F), brief publication information (G), GO analysis results (H), pie chart (I) and detailed information (J).

Curated interactions are categorized and integrated into three different interaction networks: binding interaction network, regulation network and disease association network. Publications providing experimental evidence of lncRNA binding with proteins, microRNA and transcription factors by methods such as RIP or CoIP are used to construct binding interaction network. Regulation associations based on expression experiment such as qRT-PCR or knockdown experiments are used to construct regulation network. The disease association network is constructed based on the publications in which lncRNAs are described to play validated functional roles in diseases. These three networks are assembled into the final lncRNA interaction network by merging the shared terms in their respective networks.

**Figure. 3:**
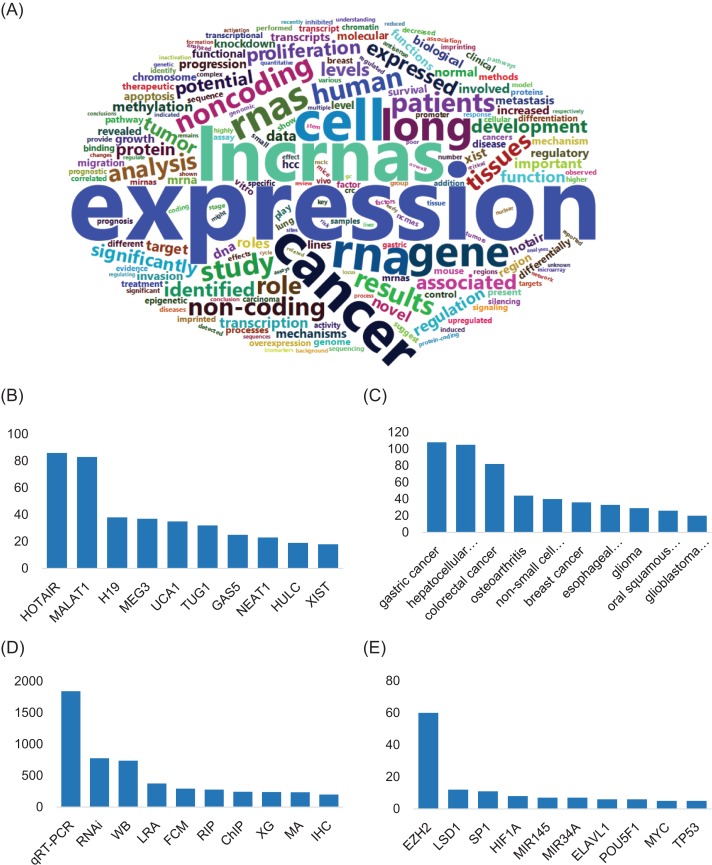
Metadata of LIVE. (A) Word cloud graph of keywords in LIVE. (B) Top 10 lncRNAs involved with lncRNA interactions ranking by number of interactions, (C) number of top 10 diseases involved with lncRNA interactions ranking by number of validated interactions evolved, (D) number of top 10 experiment type used in all interactions ranking by number of publications and (E) number of top 10 functional elements used in all interactions ranking by number of validated interactions evolved.

LIVE was developed based on the Django framework, with JavaScript for the front-end and MySQL for the back-end. The Bootstrap framework was deployed to provide compatibility for mobiles, tablets and desktop computers. To improve the visualization tools, Jbrowse (11) and echarts3 were used to provide interactive charts and graphs.

## Results

### Interface and functions

User-friendly interfaces were developed to provide direct searching and browsing (Figure 2). The search box on the main page enables users to search any keywords, including lncRNAs, proteins, genes, microRNAs and diseases. LIVE supports fuzzy searches and autocompletes with all keywords to help users investigate the results of interest. The page will redirect to the corresponding detailed page if the term exactly matches one in the database or provide all candidate results if the term partially matches terms in the database. The detailed pages for the lncRNAs consist of four sections, namely, basic information, interactive network, genomic browser and interaction details. Detailed pages for other terms, such as genes and proteins, only provide the genomic browser and interaction details. For each entry, the interactive network connected with it (represented as a node) is visualized. The node size reflects the degree of each node, indicating its importance in the interaction network. Moreover, we expanded the interaction network to include the nodes that share nodes directly connected with the entry for which the user searched. The genomic browser section contains a genomic browser for each lncRNA entry to illustrate its nearby genomic elements. For entries in the interaction details section, external links to the standard databases are provided for available terms, such as GeneCard for genes, UniPort for proteins and MalaCard for diseases. A pie button and a GO button, in addition to column labels, provide analysis results in pop-out windows. More detailed information is provided by clicking the view button, such as sample and tissue information, experimental methods, detailed pathway descriptions and publication information.

### Database metadata

Available publication abstracts were segmented to investigate the popular concerns in lncRNA studies. A large number (73%) of publications mentioned the expression patterns of lncRNAs and other genes, with `expression’ and `co-expression’ frequently noted. The regulatory functions of lncRNAs are focused on derivatives of the term `regulate’ mentioned in more than half the publications. Interestingly, `methylation’ and `proliferation’ are hot keywords, as ~35% of the analyzed publications involved those terms (Figure 3A).

**Table 1:** Comparison with other lncRNA interaction database

Database	Data type					Interaction type			Manual curation	Metadata analysis	Interactive graphs
	Disease	TF	proteins	genes	ncRNA	Binding network	Regulation network	Disease network			
LncReg	No	No	Yes	Yes	No	No	Yes	Yes	Partially	No	No
LnChrom	Yes	Yes	Yes	No	No	Yes	No	Yes	Partially	No	No
Lnc2Meth	No	No	No	No	No	No	Yes	No	Totally	No	No
LincSNP	No	No	No	No	Yes	No	Yes	Yes	No	No	Yes
EVlncRNAs	Yes	Yes	Yes	Yes	Yes	No	No	No	Totally	No	No
LIVE	Yes	Yes	Yes	Yes	Yes	Yes	Yes	Yes	Totally	Yes	Yes

LIVE also provides statistics regarding the involved functional elements and diseases in the lncRNA interaction network to facilitate research further exploring the functional roles of lncRNAs. Metadata analysis of publications illustrated preference and tendency in lncRNA studies (Figure 3B–E). A total of 83% of lncRNA-related studies are focused on tumors and cancer. The top 10 most studied lncRNAs are in 35% of the total studies. HOTAIR and gastric cancer are the most studied lncRNA and disease, respectively. The validated interactions curated from publications demonstrated that lncRNAs interact with functional elements and diseases in a very complex manner. HOTAIR, the most studied lncRNA, interacts with 26 various functional elements, such as EZH2 and PCBP1, and 40 diseases, including gastric cancer, breast cancer and colon cancer (12–16). Moreover, HOTAIR plays different roles in regulatory networks by acting in diverse ways, such as by binding with microRNAs or cis-regulating nearby genes. Additionally, 57 different lncRNAs play functional roles in gastric cancer by interacting with 42 different functional elements, including microRNAs and transcription factors. Furthermore, HOTAIR-related functional elements also interact with other lncRNAs in different tissues and diseases. Notably, 93% of lncRNAs are associated with more than one disease, and 98% are associated with more than one lncRNA. These metadata provided by our LIVE database will greatly facilitate the ability of researchers to clarify the complex interactions of lncRNAs in future studies.

## Discussion

By mining and manually curating publications to extract experimentally validated interactions, we generated LIVE, which presents the comprehensive interactions of lncRNAs with genes, proteins, ncRNAs, transcription factors and diseases in humans and mice. Moreover, LIVE provides powerful search engines and extremely conveniently interactive visualization tools. Further metadata based on the validated interactions can help researchers investigate the possible interactions with lncRNAs and explore the functional roles of lncRNAs. Comparing with similar databases, our LIVE illustrate its specialty (Table 1).

LIVE will continue to be curated and updated in the coming years, and the next update will focus on providing more kits to help analyze the metadata of the included literature.

In summary, LIVE provides a highly interactive visual database of experimentally validated interactions with lncRNAs. LIVE will facilitate the ability of researchers to investigate the potential roles and mechanisms of lncRNAs in diverse biological processes and diseases.

## Funding

Major Research Plan of the National Key R&D Program of China (2016YFC0901600); the National Natural Science Foundation of China (U1435222 and 61772543); and National Key R&D Program of China (2018YFC0910405).

*Conflict of interest*. None declared.

## Notes

**Database URL:** https://live.bioinfotech.org
